# Dermatoscopy of the Borst–Jadassohn phenomenon in hidroacanthoma simplex^☆^^☆☆^

**DOI:** 10.1016/j.abd.2019.03.004

**Published:** 2019-12-18

**Authors:** Bruno de Castro e Souza, Maria Cláudia Alves Luce, Thais do Amaral Carneiro Cunha, Neusa Yuriko Sakai Valente

**Affiliations:** a Department of Dermatology, Hospital do Servidor Público Estadual, São Paulo, SP, Brazil; b Department of Dermatology, Instituto de Assistência Médica ao Servido Público Estadual, São Paulo, SP, Brazil; c Dermatology Residency Program, Instituto de Assistência Médica ao Servido Público Estadual, São Paulo, SP, Brazil

**Keywords:** Dermoscopy, Dermatology, Poroma

## Abstract

The Borst–Jadassohn phenomenon is a morphological finding that consists of the presence of well-defined nests of cells located in the spiny stratum of an acanthotic epidermis. One of the neoplasms where this phenomenon is found is hidroacanthoma simplex. This neoplasm is considered the intraepidermal form of the eccrine poroma. Despite its benign nature, malignant transformations are reported. The present article reports a case of hidroacanthoma simplex and discusses the dermoscopy of this phenomenon.

## Introduction

The Borst–Jadassohn phenomenon is a morphological finding that consists of the presence of well-defined nests of cells located in the spiny stratum of an acanthotic epidermis. Some neoplasms, whether malignant or benign, may present this phenomenon, like as clonal seborrheic keratosis (SK), clonal Bowen's disease (BD), Paget's disease, porocarcinoma, or hidroacanthoma simplex (HS).

The distinction between these entities, both clinically and histopathologically, is sometimes a challenge. This fact can have consequences since the approach of each one is different. The dermatoscopy of the Borst–Jadassohn phenomenon has been little reported, thus it is worthwhile to describe it in HS.

## Case report

An 88-year-old male patient sought the Dermatology Service complaining of an asymptomatic lesion in the right gluteal region, with an unknown time of evolution.

**Figure 1:**
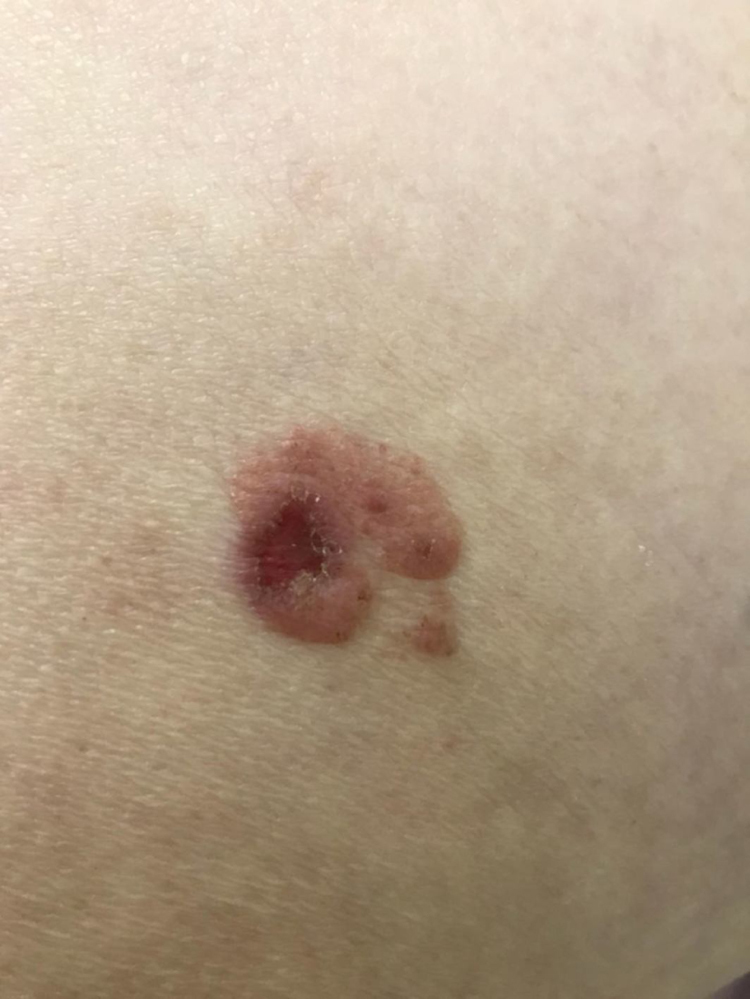
A slightly elevated pink plaque with irregular but evident borders, with a small area of exulceration.

**Figure 2:**
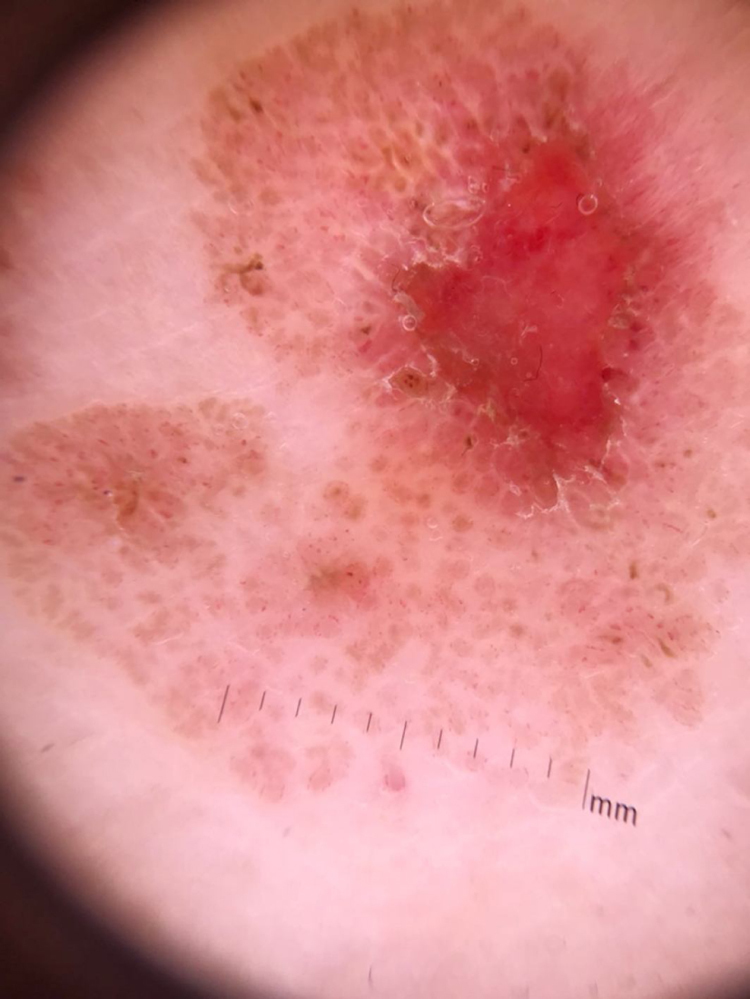
Panoramic image of the dermatoscopy showing several round, brown, well-delimited structures surrounded by dotted vessels. The exulcerated area can be seen.

**Figure 3:**
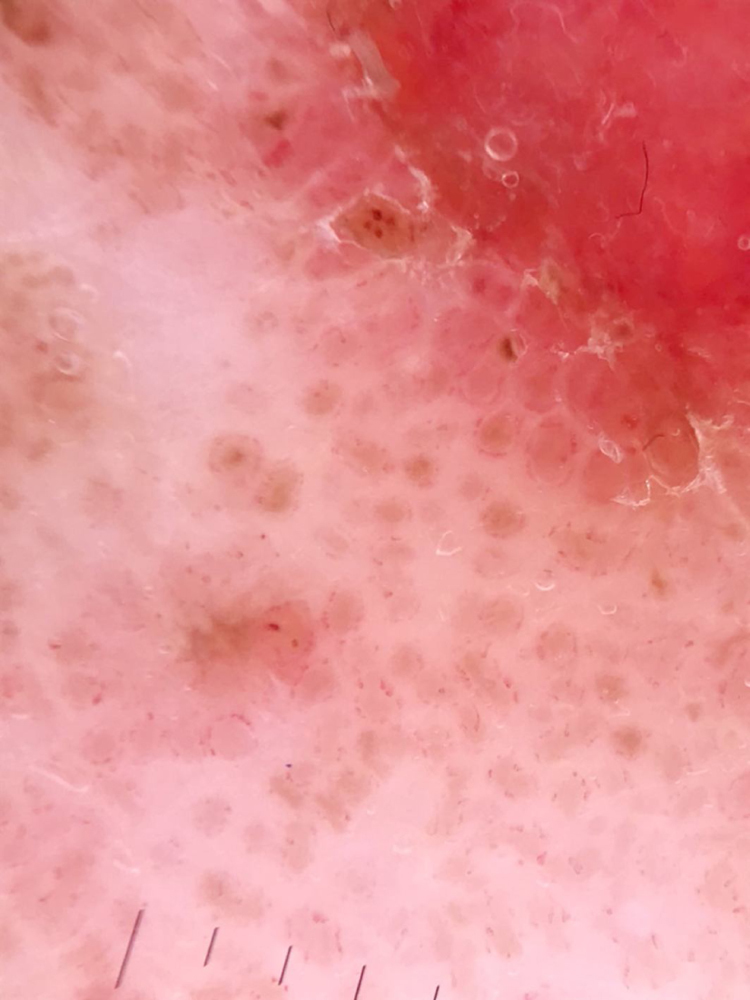
Detail of the same structures in dermoscopy.

At the dermatological examination, a slightly elevated pink plaque with irregular borders was evident, but with a small area of exulceration (Fig. 1). Dermoscopy was able to show, besides the exulceration, several well-defined brown, rounded structures, surrounded by dotted vessels (Figure 2, Figure 3).

**Figure 4:**
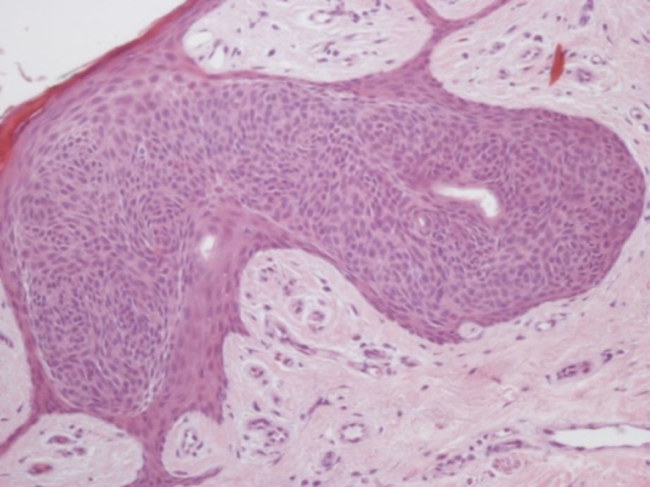
Hematoxilin & eosin, (Hematoxilin & eosin, ×200) – well-circumscribed intraepithelial proliferation of rounded and basophilic cells.

An incisional biopsy was performed with diagnostic hypotheses of seborrheic keratosis or Bowen's disease. Histopathology showed basaloid clones without atypia, in the middle of spinous cells (Fig. 4). Possibilities of HS and clonal SK were considered. The expression of carcinoembryonic antigen (CEA) and epithelial membrane antigen (EMA) in the basaloid cells and in the ductal structures present in these cells at the immunohistochemical examination indicated the diagnosis of HS.

## Discussion

In the early 20th century, Borst and Jadassohn independently described neoplasms characterized by well-demarcated cell nests located in the epidermis. Believing them to be a specific type of tumor, the name intraepithelial epithelioma of Borst–Jadassohn was coined. From the morphological and immunohistochemical studies, the majority of the authors nowadays believe that this entity represents a morphological phenomenon, rather than being specific to a certain neoplasia. Therefore, it is preferred to designate it as the Borst–Jadassohn phenomenon (BJP). This phenomenon is characterized as the presence of well-defined islands of epithelial cells, typical or atypical, within an acanthotic epidermis, and can be evidenced in some benign or malignant conditions.^1^

One of the neoplasms where BJP is found is the hidroacanthoma simplex. This neoplasm is considered the intraepidermal form of the eccrine poroma.^2^ Despite its benign nature, malignant transformations are reported in some cases, leading to the discussion regarding whether its early excision should be mandatory.^3^

Clinically, it is often mistaken for SK. This confusion may persist in histopathology and the initial diagnosis of clonal SK is not infrequent. There is a well-circumscribed intraepithelial proliferation of rounded and basophilic cells, very similar to what occurs in clonal SK that presents BJP. In the immunohistochemistry, the positivity for CEA and EMA in the cases of HS aid in the differentiation.^4^

Shiiya C et al. described four cases of HS and discussed the main dermatoscopic changes, with the central aim of distinguishing it from Bowen's disease and SK.^5^ The authors concluded that findings of small black globules (75% of cases) and fine scales distributed annularly (100%) were characteristic. In addition, the absence of glomerular vessels would help in the differentiation of DB, since such vessels found in a group are highly suggestive of the latter.

In the case reported, dotted vessels distributed in circle around rounded brown structures were observed. They represent, in the correlation of dermatoscopy with histopathology, proliferation of venules near the clonal cell blocks. Dotted vessels, when in non-melanocytic lesions, are non-specific, but the pattern found in the lesion described may be peculiar.^6^ The rounded structures represent the nests of melanocytic intraepidermal clonal cells, corresponding to BJP.

**Figure 5:**
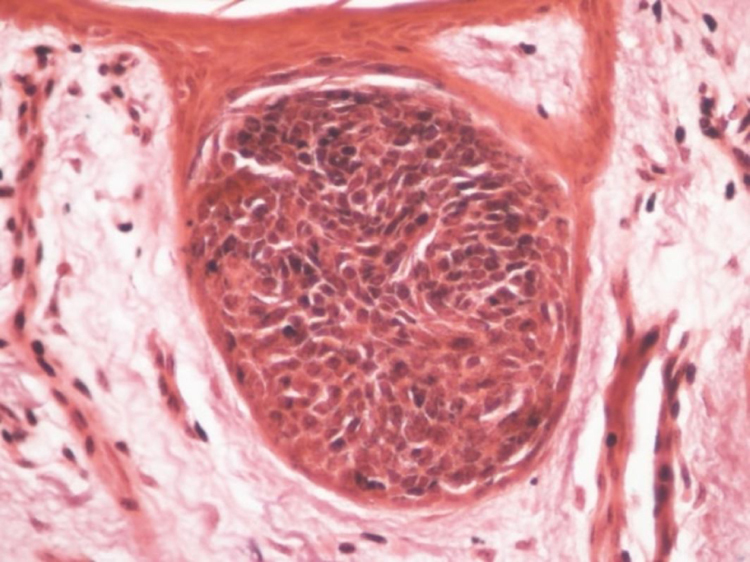
Fontana-Masson, (Fontana-Masson, ×200) – a nest of basaloid cells with a considerable amount of melanin. This phenomenon is observed in dermoscopy as the brown areas.

Although hematoxylin and eosin staining did not show the presence of pigment, when the Fontana-Masson staining was used, a great amount of melanin was observed in the blocks (Fig. 5).

In conclusion, the present study contributes to review concepts of BJP to describe the dermatoscopic alterations of this phenomenon in HS, one of the main tumors in which it develops.

## Financial support

None declared.

## Authors’ contribution

Bruno de Castro e Souza: Conception and planning of the study; composition of the manuscript; intellectual participation in the propaedeutic and/or therapeutic conduct of the studied cases; critical review of the literature.

Maria Cláudia Alves Luce: Composition of the manuscript; critical review of the literature.

Thais do Amaral Carneiro Cunha: Approval of the final version of the manuscript; conception and planning of the study; intellectual participation in the propaedeutic and/or therapeutic conduct of the studied cases; critical review of the literature.

Neusa Yuriko Sakai Valente: Participation in the design of the study; intellectual participation in the propaedeutic and/or therapeutic conduct of the studied cases; critical review of the literature; critical review of the manuscript.

## Conflicts of interest

None declared.
